# Bibliometric Analysis of Anxiety and Physical Education in Web of Science—A Performance and Co-Word Study

**DOI:** 10.3390/pediatric16040099

**Published:** 2024-12-11

**Authors:** Josué González-Ruiz, Antonio Granero-Gallegos, José-Antonio Marín-Marín, Antonio José Moreno-Guerrero

**Affiliations:** 1 Department of Didactic of Corporal Expression, Faculty of Education Sciences, University of Granada, 18071 Granada, Spain; josue@ugr.es; 2 Department of Education and Health Research Center, Faculty of Education Sciences, University of Almeria, 04120 Almeria, Spain; 3 Department of Didactics and School Organization, Faculty of Education Sciences, University of Granada, 18071 Granada, Spain; jmarin@ugr.es; 4 Department of Didactics and School Organization, Faculty of Education, Economics and Technology of Ceuta, University of Granada, 51001 Ceuta, Spain; ajmoreno@ugr.es

**Keywords:** physical education, anxiety, motivation, exercise, depression, bibliometric analysis

## Abstract

This study conducts a comprehensive bibliometric analysis of the concepts ‘physical edu- cation’ and ‘anxiety’ (PHYEDU_ANX) in the Web of Science (WoS) database. **Background/Objectives:** No previous biblio- metric studies were found that addressed this intersection, so this research is a pioneering exploration of this knowledge gap. The aim of the study is to examine the presence of both concepts in the scientific literature, identifying their trends, approaches, and future prospects. **Methods:** For this purpose, the methodology of co-word analysis was used. **Results**: The results of the study show that research on PHYEDU and ANX has traditionally focused on three main areas: motivation, exercise, and depression. In this first period, the focus was on the problem (ANX, depression…), **Conclusions**: whereas nowadays, research focuses on the subjects who suffer from it, mainly adolescents and students. The study suggests that future research in this field will focus on the areas of satisfaction, intervention, and association. This research also answers questions relevant to the field, such as which institutions or countries are the most prolific publishers of PHYEDU_ANX, as well as the most cited authors in this area of study.

## 1. Introduction

The relevance of physical education (PHYEDU) in promoting mental health is undeniable. The World Health Organization (WHO), defines mental health as a state of well-being in which the individual, aware of their own abilities, can cope with the normal stresses of life, work productively and fruitfully, and is able to make contributions to their community [1]. While not the primary focus of this study, it is important to note that PHYEDU can positively influence well-being in various ways. This is supported by research demonstrating that significant contributions of PHYEDU lead to a reduction in school bullying [2], stress [3], aggression in school context [4], helps with eating disorders by improving body self-perception [5] or increased self-esteem in students [6]. However, the present study focuses on the relationship between PHYEDU and anxiety (ANX), given the unfortunate and significant impact of ANX on society.

Numerous studies [7,8,9,10,11,12,13,14,15,16,17,18] show that regular participation in physical activity can mitigate symptoms of ANX and depression. This link is particularly significant in the educational context, where ANX often affects students’ performance and well-being. The integration of well-structured PHYEDU programs) is considered a key strategy to improving the mental health of students and teachers, and thus society as a whole [19].

Although there is a wide range of studies that address this issue, this study focuses on students’ perceptions of PHYEDU and how these vary by gender [20,21], self-perception of physical appearance [22], weight [23], age [24] or educational level [25].

Undoubtedly, the trigger for further research into intersection (PHYEDU_ANX) and its importance today was the COVID-19 pandemic. Confinement highlighted the intrinsic human need to move as an innate tool to fight ANX [26,27].

Focusing on the education sector, the disruption of face-to-face classes, and the increase in stress and ANX significantly affected students and teachers. Some studies have investigated this fact, providing a deeper understanding of how PHYEDU can adapt and alleviate ANX [28,29]. This supports findings suggesting that regular physical activity can help reduce ANX and stress levels, especially during times of crisis. Among physical education teachers, greater COVID-19 knowledge was associated with lower anxiety, particularly when workplace safety was perceived as high. Moreover, teachers who prioritized positive emotions, student relationships, and adaptable instruction reported higher levels of effectiveness and well-being during the pandemic [30,31]. Following this line, other studies [32,33] noted the importance of incorporating content on stress, ANX, and stress management into the PHYEDU curriculum—even more so when the physical activity interventions, have been shown to be effective, to help mitigate the depression and anxiety experienced by students during the pandemic [34].

PHYEDU emerged as particularly relevant during the pandemic, where educators needed tools to help students manage increased stress and ANX and increase students’ perception of safety in their environment. In this sense, the update and dynamic professional development courses for teachers are essential to adapt to the evolving needs of the educational context. Teacher workshops focused on pedagogical and health-related topics have proven effective in reducing the risk of adolescents developing elevated depressive symptoms by up to 93% [35].

Another consequence of the confinement produced by COVID-19 was the significant increase in the use of technology by the population [36,37]. Several studies analyze the transition from face to face, to online PHYEDU during the pandemic and its impact on student and teacher ANX. A study conducted during the pandemic [38] investigated the emotional self-perception of university students within virtual environments, prompted by the shift to remote learning experiences. Recurring negative emotions, such as feeling overwhelmed, stressed, and anxious were observed. These feelings are generally attributed to a sense of lost student identity due to the absence of in-person practices and interactions. The numerous benefits of physical activity, including enhanced attention and motor skills [38] may help explain why individuals unable to maintain their regular exercise routines during the pandemic experienced higher levels of depression and anxiety compared to those who remained active [39].

One more study explored the complexities associated with virtual PHYEDU and how it can generate ANX among students, providing a framework for understanding the specific dimensions of ANX in virtual learning environments [40].

At the same time, there are studies [41,42,43] that see the use of technology during the pandemic as an opportunity to innovate, increase sports practice, or reduce ANX through the use of active video games and 5G technology as a tool to improve the learning experience.

As we have seen, the scientific evidence on how PHYEDU is an effective tool to combat ANX is extensive, but on some occasions, PHYEDU becomes a trigger for ANX. This fact has not gone unnoticed by researchers, who have investigated how the assessment of physical tests may be the cause of increased ANX in students, with a special emphasis on gender perception [44,45,46]. In other studies, strategies to reduce ANX during PHYEDU classes have been investigated [47,48,49], stressing the importance of the motivational climate in the classroom, or the use of different forms of assessment, connected with this idea, it was confirmed that using ongoing formative assessment (such as quizzes or short essays) instead of a summative assessment (like a final exam) significantly reduces evaluation-related anxiety in students [50].

ANX in education is a complex and multifactorial challenge that affects both students and educators. Scientific evidence suggests that decreased socialization is associated with higher levels of ANX in students [51]. As most socialization takes place in PHYEDU classes, it is important that these programs are well designed and take into account the motivational climate and different personalities of students to mitigate the effects of ANX [52].

In the present study, a comprehensive bibliometric analysis of the concepts (PHYEDU_ANX) in the Web of Science (WoS) database is conducted [53]. Using the extensive WoS data collection, recognized for its scope and relevance in the field of education [5], the existing literature on these topics is mapped and analyzed in depth. This research is notable for its focus on the detailed assessment of the reported performance of these concepts and their representation in scholarly publications.

After reviewing the existing literature, no study has been found that addresses the intersection between “PHYEDU“ and “ANX” from a bibliometric perspective. This paper is presented as an exploratory effort to fill this knowledge gap. To this end, previous models [54,55,56] that used similar analytical techniques have been adopted as a reference, seeking to carry out robust research and contribute to filling this gap identified in the scientific field.

Consequently, the overarching goal of this study is to examine the presence of “PHYEDU” and “ANX” in WoS publications, identifying their trends, approaches, and future perspectives. The results of this research aim to highlight the importance of these concepts in the scientific literature, serving as a starting point and reference for future research in the academic community. More specifically, the specific objectives of this study are:

- To evaluate the performance of PHYEDU_ANX in the WoS scientific literature.
- To analyze the evolution of PHYEDU_ANX in the publications reported in WoS.
- To identify the key themes associated with PHYEDU_ANX in the documents indexedin WoS.
- To recognize the most influential authors in WoS who have addressed the PHYEDU_ANX concept.

After a thorough evaluation of the implications related to PHYEDU_ANX, it is clear that this field offers a vast and valuable set of findings. Studies have consistently shown that PHYEDU plays a crucial role in promoting mental health and well-being by mitigating symptoms of ANX and depression, particularly in educational settings. The integration of structured PHYEDU programs is recognized as an essential strategy to improve the mental health of students and teachers, thus contributing to a healthier society.

The interaction of these two fields (PHYEDU_ANX) suggests a rich and complex area of research, where a bibliometric analysis can provide valuable contributions to society, in general, and to the education system in particular.

## 2. Materials and Methods

### 2.1. Research Design

The study employs a bibliometric approach to research. This methodology stands out for its ability to comprehensively quantify and analyze the scientific literature [57]. Furthermore, the proposed research design facilitates the searching, recording, analysis and prediction of the scientific literature concerning current knowledge [58]. In particular, this study was based on the co-word analysis methodology [59], which is based on the analysis of keywords extracted from publications. This technique not only makes it possible to identify the interrelationships among several topics in the scientific literature but also has the capacity to project possible emerging areas for future research. This technique makes it possible to visualize, in the form of maps, the junctions that reflect the performance, the location of conceptual subdomains, and the evolution of knowledge in the area of study. In addition to this technique, analyses of various detailed bibliometric parameters were performed, including indices such as h, g, hg, and q2 [60], which provided a deeper insight into the impact and relevance of the analyzed papers in the field of study.

### 2.2. Procedure

To ensure the integrity and objectivity of the study, an approach based on previous impact research was followed [61,62]. The methodological process involved different stages: in the first stage, Web of Science (WoS) was selected as the ideal database to carry out the documentary analysis. In the second, the concept of interest (PHYEDU_ANX) was defined to address the relevant scientific literature. Then, a specific search equation (“physic* education*” (Topic) AND “anxiety*” (Topic) was developed using the titles of the indexed documents as a search criterion—525 publications were found. Next, in the third phase, the search was narrowed down to the WoS core compilation, focusing on the indexes SCI-EXPANDED, SSCI, A&HCI, CPCI-S, CPCI-SSH, BKCI-S, BKCI-SSH, ESCI, CCR-EXPANDED, and IC. Finally, the inquiry range was defined as 1976 to 2022 inclusive. Strict exclusion criteria were applied, eliminating duplicates, incorrectly indexed items, and documents after 2022, since the year 2023 had not yet ended. These procedures resulted in a final unit of analysis of 481 documents.

**Figure 1:**
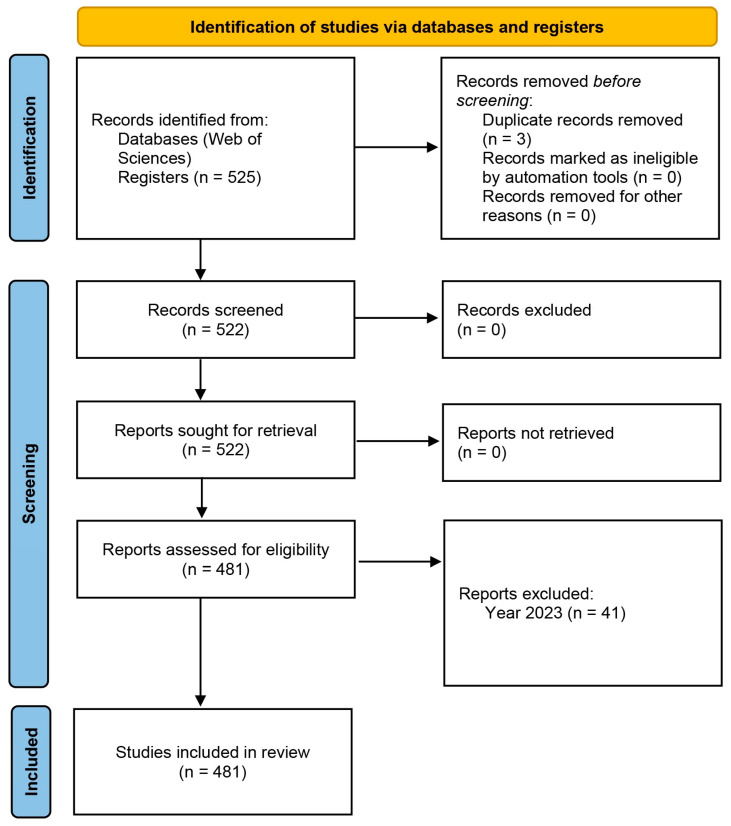
Flowchart according to the PRISMA declaration.

On the other hand, the guidelines established by the PRISMA 2020 declaration [63] were implemented, with the aim of following a standardized scientific inquiry procedure. The process was documented through a flow chart (Figure 1).

### 2.3. Data Analysis

With the aim of analyzing the bibliometric characteristics of the documents, the Analyze Results and Creation Citation Report software (SciMAT) were used. These allowed the examination of variables such as: year of publication, authorship, country of origin, document type, institution, language, publication medium, and number of citations received on the COMPU-EDU. To ensure the most relevant findings, specific inclusion criteria were implemented, focusing on the year of publication (encompassing the whole of the literature from 1976 to 2022); language (x ≥ 9); area of knowledge (x ≥ 20); type of documents (x ≥ 20); organizations (x ≥ 9); authors (x ≥ 5); sources of origin (x ≥ 10); countries (x ≥ 30); the three most cited documents (x ≥ 30); and the three most cited documents (x ≥ 10).

Without the application of these criteria, the visualization of results in tables would become impractical. The massive number of resulting cells would hinder interpretation and analysis. As a result, only publications meeting these criteria are included in the results tables.

Furthermore, this research employed a methodological approach grounded in the longitudinal analysis of information extracted from the collected documents. Utilizing SciMAT, the dynamic and structural development of the units of analysis was explored, enabling the mapping of keyword evolution into distinct topics across the defined temporal intervals. Information processing was similarly conducted through SciMAT:

Recognition: Keyword analysis publications (n = 19,919) served as the foundation for the development of co-occurrence maps, illustrating the relationships between key terms. Next, a standardized interconnected co-occurring terms was constructed and the keywords with the highest significance were identified (n = 18,642). A clustering algorithm was instrumental in uncovering the most significant concepts and themes.

**Figure 2:**
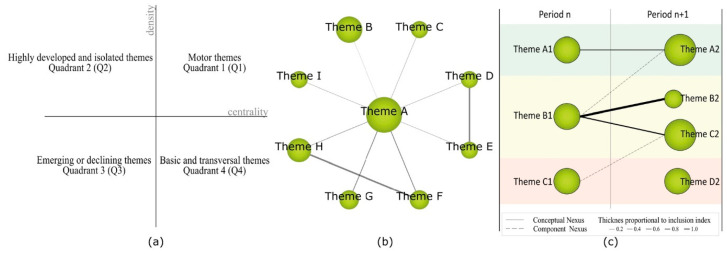
Strategic diagram (**a**). Thematic network (**b**). Thematic evolution (**c**) (Herrera-Viedma et al., 2020) [65].

Reproduction: Terms were weighted according to their significance in the literature and visualized through strategic diagrams. These diagrams were then classified (Figure 2a) and were systematically partitioned into four distinct quadrants (Q), with the first quadrant (Q1) positioned in the upper right = driving and relevant themes; positioned in the upper left (Q2) = entrenched or isolated themes; lower left (Q3) = themes with escalating or dwindling relevance to the field; lower right (Q4) = themes that are underdeveloped or cross-cutting. In the construction of the networks, the principles of density and centrality were applied. Density was measured as the internal cohesion of each network, while centrality was evaluated based on the degree of interconnection between the different networks. The topics addressed in the publications served as the basis for the configuration of these networks [64] (Figure 2b). These networks map the connections between the main topic and related terms.

Determination: In order to analyze the evolution of nodes over time, the publications were categorized into distinct time periods. This process involved establishing four specific periods for analysis (P1 = 2000–2004; P2 = 2005–2009; P3 = 2010–2014; P4 = 2015–2020). Time periods were determined based on documentary similarity, except for author analysis, which considered the entire study timeframe as a single period (PX = 2000–2020). The degree of association between the time periods was determined by the quantity of shared keywords or themes.

Performance: Output indicators were chosen for this process based on specific inclusion criteria (Table 1). The analysis also considered the evolving significance of themes across the established periods (Figure 2c).

## 3. Results

### 3.1. Performance and Scientific Output

**Figure 3:**
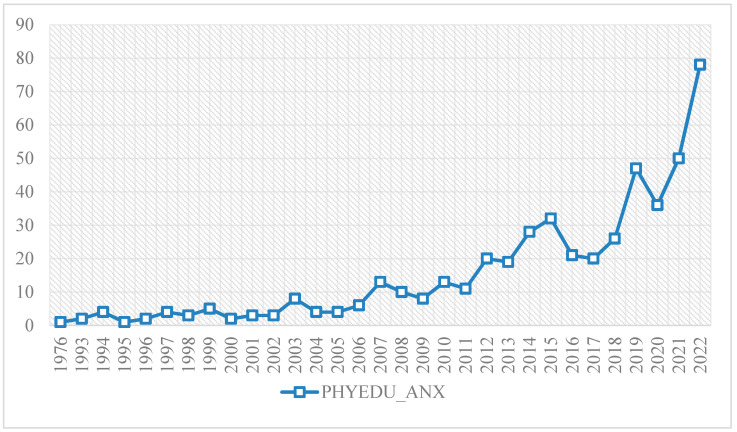
Evolution of scientific production.

The scientific production of PHYEDU_ANX in WoS is 481. This production has been uneven since the beginning of its records in WoS. The first paper in this database dates back to 1976. From that date until 1993, no papers were produced on the research topic. From 1993 to 2022, the production has been constant in date, although uneven in volume of production. From 1993 to 2006, the number of manuscripts ranged from one to eight. It is from 2007 onwards that the volume starts to increase more considerably. This increase from 2007 onwards has different trends. From 2007 to 2015, there was an upward trend in the volume of production. In 2016, there was a sharp drop in production, which remained constant until 2018. In 2019, there was another sharp increase, although in 2020 there was a slight drop again. Finally, from 2021 to 2022, the volume increased substantially; so much so that both years are the two years with the highest volume of scientific production on PHYEDU_ANX. In 2021, 50 manuscripts were written and in 2022, 78 manuscripts were written (Figure 3).

English can be considered the language par excellence in manuscripts on the subject of PHYEDU_ANX. The other languages have a much lower volume of production (Table 2).

The areas of knowledge that focus on the field of study of PHYEDU_ANX are mainly “Education Educational Research” and “Sport Sciences”. There are other areas, such as hospitality and psychology, which also cover part of this type of study (Table 3).

Researchers mainly use research articles to present their results. This is followed, with a very low volume of production compared to research articles, by conference papers (Table 4).

European institutions are pioneers in this field of study. UDICE, from France, stands out, followed by the Universities of Almeria and Granada, respectively. There are no significant differences between the leading institutions in terms of volume of production (Table 5).

In the PHYEDU_ANX field of study, all authors demonstrate a comparable level of influence, with no single individual dominating the field in terms of volume of production. In this case, the scientific production is even. The authors “Barkoukis, V.” and “Jaakkola, T.” stand out for having a total of seven manuscripts on the subject. They are followed by “Robazza, C.”, with six productions (Table 6).

In relation to publication, sources show equal productivity of journals that publish research articles. The journal “Psychiatria Danubia” stands out with a total of fourteen manuscripts (Table 7).

Both the United States and Spain, in that order, stand out as the countries with the highest volume of production on the PHYEDU_ANX theme (Table 8).

In the PHYEDU_ANX field of study, the citation volume of the most cited manuscript does not exceed 1000. The manuscript with the highest volume of production is the one by Gilbody et al. [66], with a total of 546. This manuscript is a systematic review of educational plus organizational interventions for enhancing depression management. The next highest ranked manuscripts have approximately half the number of citations (Table 9).

### 3.2. Structural and Thematic Development

**Figure 4:**
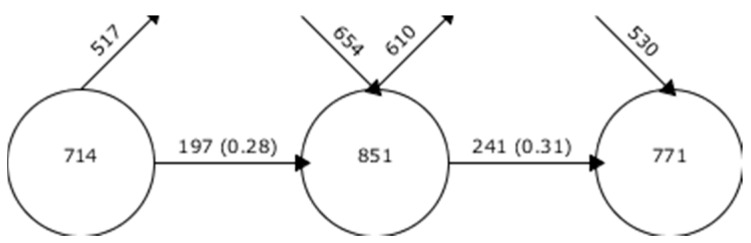
Continuity of keywords between contiguous intervals.

The analysis of the keywords reveals multiple aspects: firstly, the number inside the circle quantifies the total terms used by authors during a particular period. Secondly, it shows the keywords that have ceased to be used in the following period, symbolized by an ascending arrow; the new keywords introduced in the same inter-value, indicated by a descending date; and finally, the keywords that are reused between periods, shown by a horizontal date. The data presented in Figure 4 reveal a number of facets that need to be highlighted and analyzed in depth. In this case, it is observed that each period contains an equivalent number of keywords. The percentage of coincidence between periods is increasing. Between the first period and the second period it is 28%, while between the second period and the third period it is 31%. This indicates that there is a well-established research base in the scientific community, which, as the research progresses, further strengthens the subject of study.

**Figure 5:**
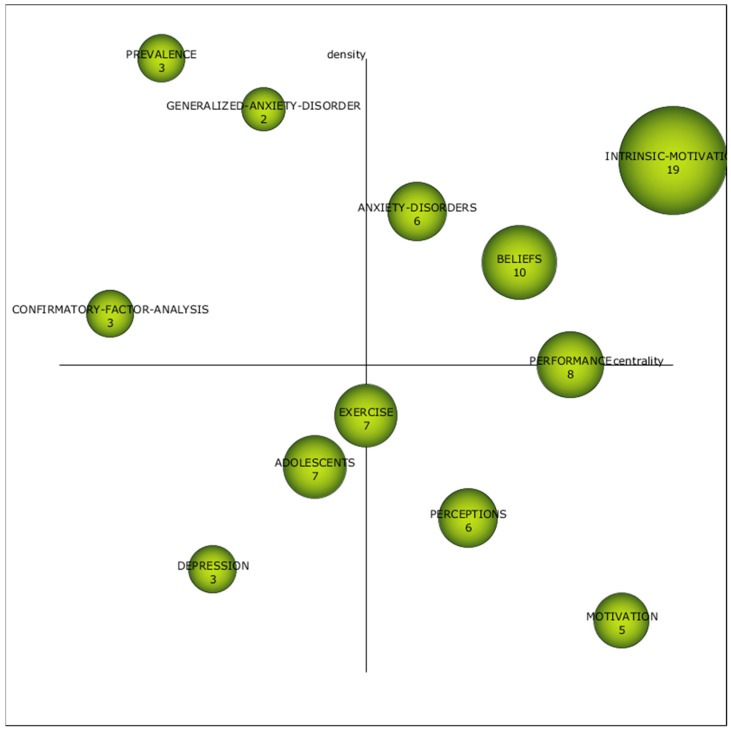
Interval diagram as the academic performance of the subjects derived from the co-word analysis of the first period (1976–2013).

This analysis examines the inter-value diagram and assesses the academic performance of themes extracted via co-word analysis. The inter-value diagram reveals the degree of importance of the themes using Callon’s index (Figure 5), employing a clustering approach that leverages centrality and density. Specifically, centrality measures how strongly a theme is linked to others, while density is based on the intensity of the external relationships in each theme. On the other hand, academic performance provides the bibliometric value of the various subjects analyzed, using indicators such as the h-index, g-index, hg-index, q2-index and the average number of citations per document.

In the first period established for the analysis, between 1976 and 2013, inclusive, the subject “intrinsic-motivation” is the one with the highest academic performance, given that its bibliometric values are higher than the rest. This theme is also situated as a driving theme in the diagram, together with the themes “anxiety-disorders” and “beliefs”. Therefore, it can be established that these themes mark the main research trends in this period. If we focus on the driving themes of this period, we can see that the “intrinsic-motivation” theme focuses on “physical-education”, “hierarchical-model”, “trait-anxiety”, “achievement-goals”, “goal-orientations”, “orientations”, “sport” and “strategies”; the “anxiety-disorders” theme focuses on “panic-disorder”, “major-depression”, “ranch”, “major-depression”, “ranch” and “major-depression”; “major-depression, randomized-controlled-trial, mental-disorders, physician-education, health-care and psychotherapy; and the beliefs theme focuses on task, ego-orientation, emotions, experiences, efficacy, high-school, success and ability. It can be established that the analysis identifies the theme of “intrinsic-motivation” as encompassing areas such as PHYEDU, hierarchical model, trait ANX, achievement goals, goal orientations, sport and strategies. On the other hand, the theme of “anxiety disorders” focuses mainly on panic disorder, major depression, randomized controlled clinical trials, mental disorders, physician education, healthcare, and psychotherapy. Finally, the “beliefs” theme relates to concepts such as homework, selfish orientation, emotions, experiences, efficacy, high school, success, and ability (Figure 5).

**Figure 6:**
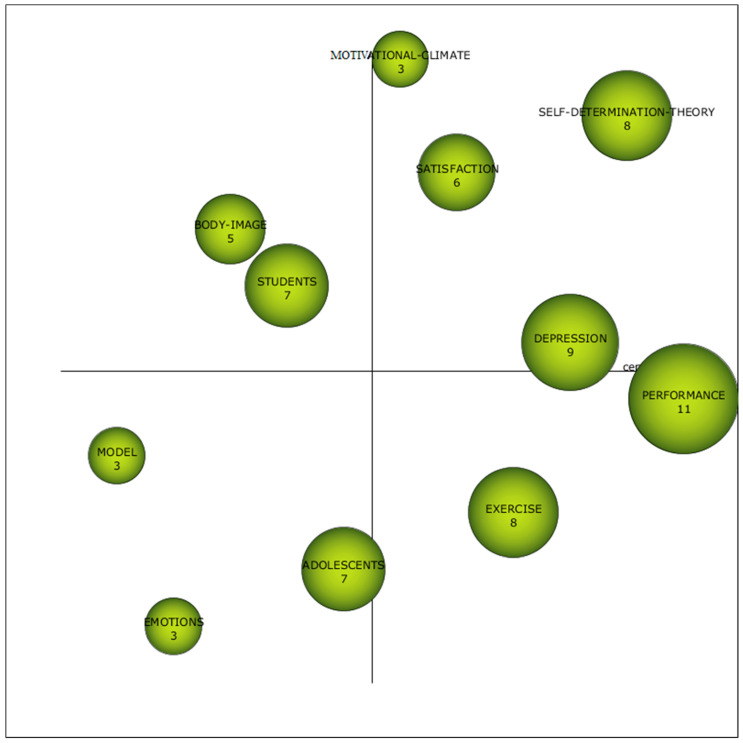
Interval diagram as the academic performance of the subjects derived from the co-word analysis of the first period (2014–2019).

In the second established period, from 2014 to 2019, a subject with a higher academic performance is observed. This is the case of “performance”. In this period, the difference with the rest of the subjects is not as high as in the previous period. In this case, it is closely followed by “depression”, “exercise”, and “self-determination-theory”. As can be seen, the subjects with the highest academic performance vary with respect to the previous period. The same applies to the driving themes, which in this case are “self-determination-theory”, which focuses on “need-satisfaction”, “autonomy-support”, “academic-motivation”, “extrinsic-motivation”, “social-physique-anxiety”, “participation”, “intrinsic-motivation” and “subjective-vitality”; “satisfaction”, which focuses on “engagement”, “outcomes”, “mediating-role”. It can be established that self-determination-theory addresses concepts such as need satisfaction, autonomy, academic motivation, social ANX, participation, intrinsic motivation, and subjective vitality. Satisfaction-theory focuses on engagement, outcomes, mediating role, behavior, self-efficacy, property-psychometric, achievement, and English. The “motivational-climate” theme covers perceived competence, success, football, achievement goals, coach, players, skill, and sport. Finally, the “depression” theme relates to stress, mental health, climate, disorders, teachers, ANX, substance use and, prevalence (Figure 6).

**Figure 7:**
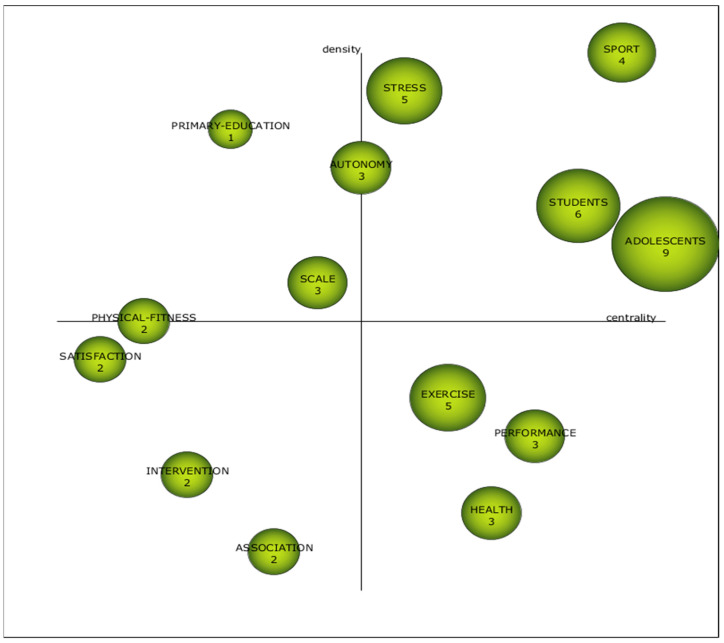
Interval diagram as the academic performance of the themes derived from the co-word analysis of the first period (2020–2022).

In the last established period, between 2020 and 2022, the theme “adolescents” emerges as the highest academic performance, with a marginal lead over other themes resulting from the analysis carried out. During this period, the driving themes are “stress”, which is related to “pain”, “young-adults”, “heart-rate-variability”, “risk-factors”, “randomized-controlled-trial”, “COVID-19”, “bruxism” and “temporomandibular-disorders”; “sport” which focuses on “high-performance”, “celebrity”, “autobiography”, “experiences”, “childhood”, “physical-education”, “motivational-climate” and “sports-pressure”; “students’, which focuses on ‘emotional-intelligence’, ‘happiness’, ‘sedentary-behaviour’, ‘anxiety’, ‘psychometric-properties’, ‘gamification’, ‘COVID-19-related-anxiety’, ‘subjective-well-being’; and ‘adolescents’, which relates to ‘children’, ‘mental-health’, ‘yoga’, ‘depressive-symptoms’, ‘disorders’, ‘physical-activity’, ‘depression’ and ‘young-people’. The study highlights several key themes. Firstly, stress, related to pain, young adults, heart rate variability, risk factors, randomized controlled clinical trial, COVID-19, or bruxism. The second theme focuses on sport, exploring aspects such as high performance, celebrities, autobiography, experiences, childhood, PHYEDU, motivational climate, and sport pressure. The third theme focuses on students, addressing topics such as emotional intelligence, happiness, sedentary behavior, ANX, psychometric properties, gamification, COVID-19-related ANX, and subjective well-being. Finally, the fourth theme, linked to adolescents, deals with aspects such as children, mental health, yoga, depressive symptoms, disorders, physical activity, depression, and young people. Finally, it can be indicated that the themes “satisfaction”, “intervention”, and “association” merit ongoing observation in the coming years, given their placement in the diagram, as they could evolve into influential themes or disappear from the field of study of PHYEDU_ANX (Figure 7).

Themes positioning within Figure 3, Figure 4, Figure 5 and Figure 6 are shown in Table 10. The data show that there are three themes that appear in all periods. This is the case for “performance”, “exercise” and “adolescents”. This shows that there is no conceptual gap, and therefore, there is a study base established in PHYEDU_ANX, whether or not they have a greater or lesser weight in the periods analyzed. For example, it is observed that the theme “performance” is established as a driving theme in P1, while in the rest of the periods, it loses relevance. On the other hand, “adolescents” is less relevant in the first two periods, while in the last period it is considered a driving theme (Table 10).

**Figure 8:**
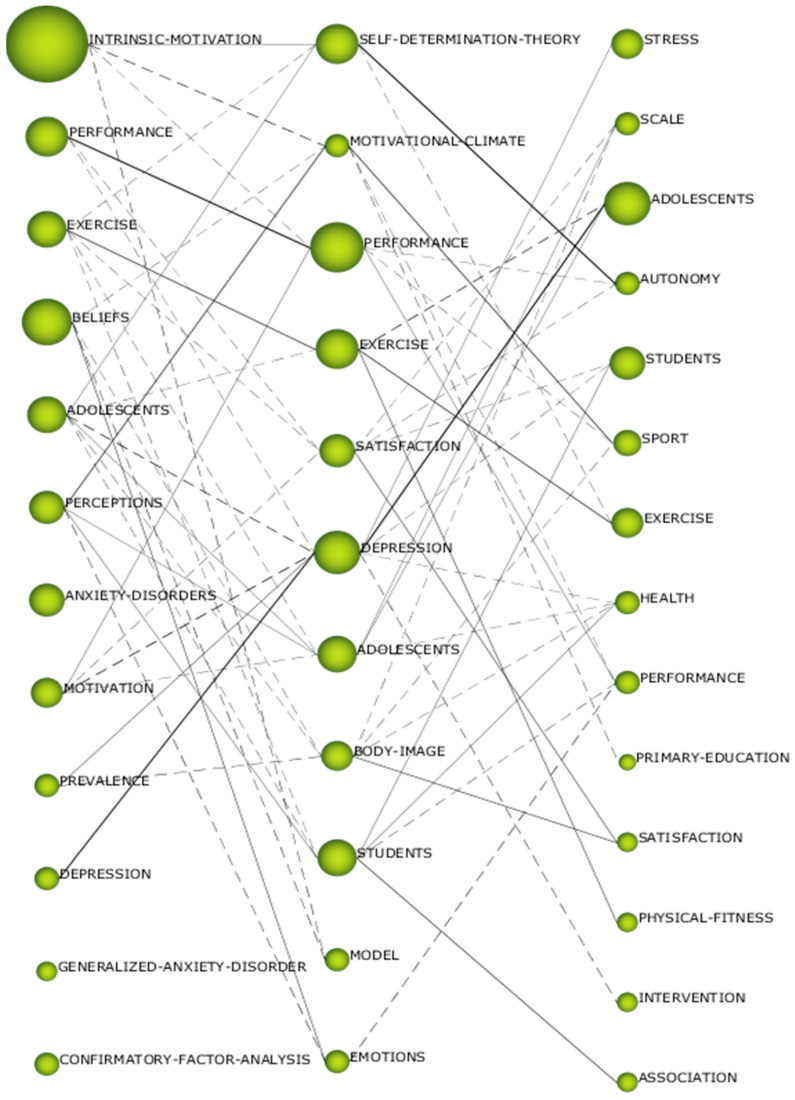
Thematic evolution by h-index.

The thematic evolution of the studies related to PHYEDU_ANX is determined by the Jaccard index (Figure 8). The connection between topics is established according to the presence of keywords or shared themes. The representation of these connections varies according to the type of relationship: keyword-based connections are illustrated with dashed lines, also called non-conceptual connections, while theme-based connections are represented with solid lines, known as conceptual connections. In addition, the thickness of the line varies according to the number of coincidences between themes in consecutive periods, with a greater thickness indicating a greater number of coincidences in terms of themes or keywords. Bearing in mind the data given in Figure 8, several lines of research stand out from the rest. These are the cases of “intrinsic-motivation, self-determination-theory_autonomy”, “perceptions_motivational-climate_sport”, “exercise_exercise_exercise” and “depression_depression_adolescents”. This indicates that there are several established lines of research in the PHYEDU_ANX field of study, focusing on motivation, exercise, and depression. It also shows that the scientific community has given importance to these lines of research in this field of study. It should also be noted that there is a greater number of conceptual than non-conceptual connections, which shows a strong relationship between the established lines of research. In other words, the various lines of study are related to a greater or lesser extent to each other.

### 3.3. Authors with the Highest Relevance Index

**Figure 9:**
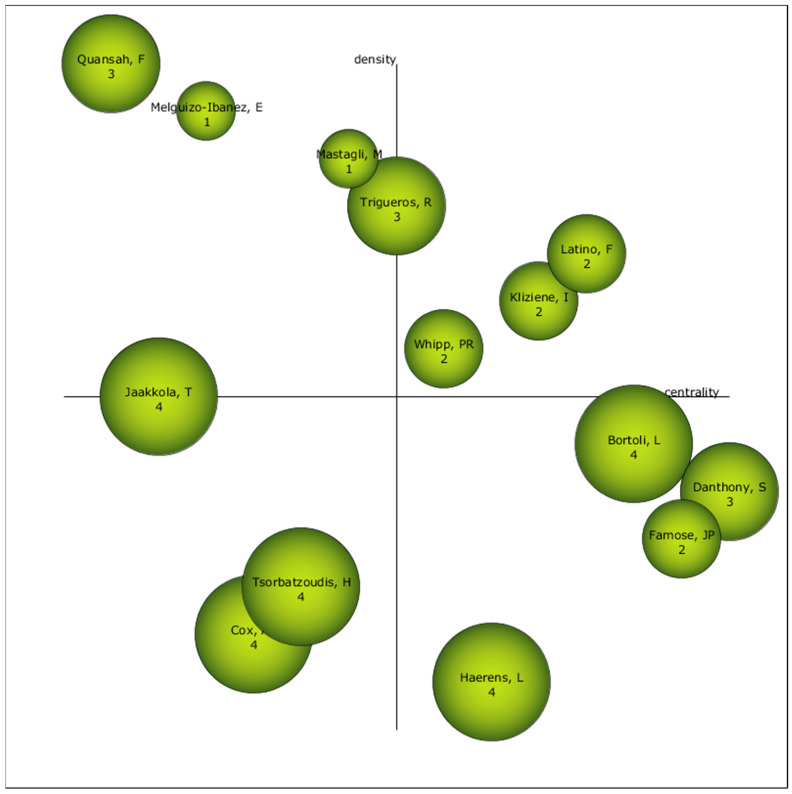
Evolution of authors by h-index.

With respect to the authors, three stand out as driving authors. These are Latino, F., Kliniene, I., and Whipp, PR. In addition, the authors Tsorbatzoudis, H., and Cox, AE, who are placed in the diagram as authors who may become, in the near future, driving authors in the field of study of PHYEDU_ANX (Figure 9).

## 4. Discussion

It is important to note that this bibliometric study is a pioneer in the analysis of scientific production in PHYEDU_ANX, so we will not be able to compare the results obtained with other previous or contemporary studies.

This study aimed to determine the presence of both concepts in the scientific literature, identifying their trends, approaches and future perspectives. The results obtained indicate that the scientific production in PHYEDU_ANX has shown a clear evolution over time, starting sporadically in 1976 and experiencing significant fluctuations up to the present. The variability in production may be due to multiple factors, including changes in funding, evolving scientific interests, and response to emerging societal needs.

Two points of upward inflection can be observed: the first starting in 2007, with a notable increase in production that may be linked to a greater recognition of the importance of this area of study or to methodological advances that have facilitated research [69]. The second is a consequence of the COVID-19 pandemic, which had a notable impact on the increase in ANX and the importance of PHYEDU [70,71]. Thus, the years 2021 and 2022 were the years with the highest scientific production in the entire period analyzed.

The analyses of linguistic, geographical, and institutional leadership reveal that English has consolidated as the main language in the scientific production of PHYEDU_ANX, especially in databases such as WoS. This trend reflects the position of English as the lingua franca of science, but also raises questions about accessibility and diversity in scientific communication. It should be noted here that Spanish is positioned as the second scientific language in this field of research, confirming its role as the second most spoken mother tongue in the world and the fourth in total number of speakers [72]. Continuing with the European theme, it is the universities of southern Europe that occupy the top places in this field of research, with the UDICE, from France, and the Universities of Almeria and Granada, from Spain, standing out. In terms of research output, Spain is ranked as the second-largest scientific producer worldwide in this field of research, only surpassed by the United States.

In the Authors and Scientific Production category, we can underline the authors “Barkoukis, V.” and “Jaakkola, T.” These are the authors with the highest volume of production in the PHYEDU_ANX field of study. However, it is the authors Latino F., Kliniene, I., and Whipp, PR who are considered most relevant in this field of study. In addition, Tsorbatzoudis, H., and Cox, AE, who may become the most relevant authors in this field of study in the near future, should be kept in mind in the coming years.

Regarding the impact and relevance of publications, the manuscript with the highest volume of production is the manuscript written by Gilbody et al. (2003) [52], with a total of 546 citations. This article carries out a systematic review of educational and organizational interventions to improve the management of depression. This shows the great value of this type of study in the scientific field. There is no journal that stands out considerably above the rest in terms of volume of production. The journal “Psychiatria Danubia” is the journal with the highest volume of production, with a total of 13 manuscripts. The constant appearance and evolution of keywords and topics reflects a well-established and continuously evolving study base.

The consistent themes and their evolution show that as the research progresses, the level of coincidence increases, which determines the coincidence of the authors when it comes to delimiting the lines of research in this field of study. In relation to the analysis of academic performance, the results show how several main ideas evolve with the periods of production. In the first period (1976 to 2013) the motor themes “Intrinsic-motivation” are identified, which focuses on areas such as PHYEDU, hierarchical model, trait ANX, achievement goals, goal orientations, sport and strategies. “Anxiety-disorders” focuses on panic disorder, major depression, randomized controlled clinical trials, mental disorders, medical education, medical care, and psychotherapy. “Beliefs” relates to concepts such as homework, ego orientation, emotions, experiences, efficacy, high school, success, and ability. In the second analysis period (2014 to 2019), the top performing themes are “performance,” followed closely by “depression,” “exercise,” and “self-determination-theory,” while the top performing motor themes are “self-determination-theory,” “satisfaction,” “motivational-climate,” and “depression.” In the last period (2020 to 2022), the theme “adolescents” emerges as the one with the highest academic performance, while the driving themes during this period are “stress,” which addresses aspects such as pain, young adults, heart rate variability, risk factors, randomized controlled clinical trials, COVID-19 and bruxism. Throughout all the periods analyzed, three themes remain consistent: “performance,” “exercise,” and “adolescents.”, indicating that there is no conceptual gap in the PHYEDU_ANX field of study across the periods analyzed. The constant presence of these themes suggests a consolidated study base in PHYEDU_ANX, regardless of whether they have greater or lesser weight in the specific periods. Once the state of the art in this field of research, its trends and approaches have been analyzed, we will finish answering the main objective of the study by examining the possible motor themes. The analysis emphasizes the importance of the themes “satisfaction,” “intervention,” and “association” for future research as possible driving terms in the future, confirming the relevance that several authors give to the elaboration of educational interventions in PHYEDU [38] in order to act more effectively against ANX. This analysis not only reflects the current state of the field of PHYEDU_ANX, but also provides insights for future research and educational interventions.

## 5. Conclusions

The discussion highlights the complexity and dynamism of the PHYEDU_ANX field of study. The scientific production, although fluctuating, shows a general tendency to increase, especially after the confinement as a consequence of COVID-19, with a consolidation of certain themes such as motivation, exercise, and depression. In the first period of analysis the focus was on the problem (ANX, depression…), while at present, research is focusing on the subjects who suffer from it, mainly adolescents and students.

Leading institutions and authors reflect a competitive and collaborative landscape, while the impact and relevance of certain publications underline the importance of applied research in this field. The analysis of the scientific literature consolidates the scientific evidence that PHYEDU is an effective tool for combating ANX. The study suggests that future research in this field will focus on the areas of satisfaction, intervention, and association.

## Figures and Tables

**Table 1:** Production indicators and inclusion criteria.

Configuration	Values
Analysis unit	Keywords authors, keywords Web of Science (WoS)
Frequency threshold	Keywords: P1 = (2), P2 = (2), P3 = (2)
	Authors: PX = (2)
Network type	Co-occurrence
Co-occurrence union value threshold	Keywords: P1 = (2), P2 = (2), P3 = (2)
	Authors: PX = (2)
Normalization measure	Equivalence index: eij = cij2/Root (ci − cj)
Clustering algorithm	Maximum size: 9; Minimum size: 3
Evolutionary measure	Jaccard index
Overlapping measure	Inclusion rate

**Table 2:** Scientific language used.

Languages	n
English	420
Spanish	35
Russian	10

**Table 3:** Areas of knowledge.

Denomination	n
Education Educational Research	124
Sport Sciences	102
Hospitality Leisure Sport Tourism	85
Psychology Applied	47
Psychology	44

**Table 4:** Document type.

Denomination	n
Article	409
Proceedings Paper	33
Meeting Abstract	23

**Table 5:** Institutions.

Denomination	n
UDICE French Research Universities	13
University of Almería	11
University of Granada	11
Aristotle University of Thessaloniki	10

**Table 6:** Most prolific authors.

Authors	n
Barkoukis, V.	7
Jaakkola, T.	7
Robazza, C.	6

**Table 7:** Source of provenance.

Journals	n
Psychiatria Danubia	14
European Physical Education Review	13
Retos	13
International Journal of Environmental Research and Public Health	11
Sustainability	11

**Table 8:** Country of origin.

Countries	n
USA	78
Spain	65
China	45
Turkey	38
Australia	33
United Kingdom	31

**Table 9:** Most cited articles.

Reference	Citations
Gilbody, S., Whitty, P., Grimshaw, J., and Thomas, R. (2003). Educational and organizational interventions to improve the management of depression in primary care—A systematic review. JAMA-Journal of the American Medical Association, 289(23), 3145–3151. https://doi.org/10.1001/jama.289.23.3145	546
Katzelnick, DJ, Simon, GE, Pearson, SD, Manning, WG, Helstad, CP, Henk, HJ, Cole, SM, Lin, EHB, Taylor, LH, and Kobak, KA (2000). Randomized trial of a depression management program in high utilizers of medical care. Archives of Family Medicine, 9(4), 345–351. https://doi.org/10.1001/archfami.9.4.345	279
Aktekin, M., Karaman, T., Senol, YY., Erdem, S., Erengin, H., and Akaydin, M. (2001). Anxiety, depression and stressful life events among medical students: a prospective study in Antalya, Turkey. Medical Education, 35(1), 12–17. https://doi.org/10.1046/j.1365-2923.2001.00726.x	257

References: [66,67,68].

**Table 10:** Principal research themes related to PHYEDU_ANX from 1976 to 2022.

	P1 (1976–2013)	P2 (2014–2019)	P3 (2020–2022)
Intrinsic-motivation	Q1 (130.79–42.82)		
Performance	Q1–Q4 (44.94–22.99)	Q4 (69.88–12.7)	Q4 (37.99–8.59)
Exercise	Q3–Q4 (24.3–21.86)	Q4 (37.96–10.95)	Q4 (31.49–10.27)
Beliefs	Q1 (44.43–36.02)		
Adolescents	Q3 (20.47–20.53)	Q3 (31.16–7.58)	Q1 (70.48–24.06)
Perceptions	Q4 (39.03–16.28)		
Anxiety-disorders	Q1 (33.33–39.5)		
Motivation	Q4 (47.56–5.17)		
Prevalence	Q2 (1.05–82.86)		
Depression	Q3 (2.04–15.48)	Q1 (48.42–16.43)	
Generality-anxiety-disorder	Q2 (13.33–77.78)		
Confirmation-factor-analysis	Q2 (0–25)		
Self-determination-theory		Q1 (56.75–35.37)	
Motivation-climate		Q1 (34.18–36.27)	
Satisfaction		Q1 (37.48–18.66)	Q3 (3.06–14.81)
Body-image		Q2 (21.86–17.75)	
Students		Q2 (29–86-17–57)	Q1 (41.22–29.83)
Model		Q3 (4.11–11.11)	
Emotions		Q3 (5.37–6.67)	
Stress			Q1 (30.43–43.21)
Scale			Q2 (23.22–21.48)
Autonomy			Q1-Q2 (24.7–30.94)
Sport			Q1 (49.27–99.26)
Health			Q4 (33.71–6.75)
Primary-education			Q2 (12.59–35)
Physical-fitness			Q2-Q3 (6.56–20)
Intervention			Q3 (7.94–7.62)
Association			Q3 (17.18–5.93)

## Data Availability

The data are available in the specialized database Web of Science, and in the study, for any further information please contact the reference author by e-mail.
